# Adverse Reactions to Sulphasalazine

**Published:** 1987-08

**Authors:** M. Hamilton, A. J. K. Williams, M. J. Hall

**Affiliations:** Senior House Officer Department of Medicine, Bristol Royal Infirmary; Research Fellow Department of Medicine, Bristol Royal Infirmary; Lecturer Department of Medicine, Bristol Royal Infirmary


### Adverse Reactions to Sulphasalazine

**M. Hamilton, BM, ChB**
Senior House Officer
**A. J. K. Williams, BSc, MRCP**
Research Fellow
**M. J. Hall, MSc, MRCP**
Lecturer
Department of Medicine, Bristol Royal Infirmary

### INTRODUCTION

Sulphasalazine is a drug which is the therapeutic mainstay in the prevention of recurrence of ulcerative colitis and is also used in the treatment of active disease. It consists of a sulphonamide (sulphapyridine) and a salicylate (5-amino salicylic acid) component with the latter being the active moiety in colitis. However it was originally developed as a treatment for rheumatoid arthritis (1) and in the past 3 years there has been a renaissance both locally and nationally in its use by rheumatologists as a second line agent in the treatment of rheumatoid arthritis (2,3) and to a lesser extent in ankylosing spondylitis. It is regarded as a safe drug when compared with the severe side effects reported with the other commonly used disease-modifying agents in rheumatoid arthritis such as gold and penicillamine.

We would like to add a note of caution to this view of sulphasalazine by reporting three cases of severe neutropenia in patients which occurred over a four month period between 1983 and 1984 in the Bristol Royal Infirmary in association with this drug.

### CASE 1

A 63 year old retired barmaid with active rheumatoid arthritis was treated with sulindac 400 mg daily, chlormezanone 200 mg nocte and sulphasalazine 1 g daily. Haemoglobin was 11.2 g/dl and neutrophil count was $5.3 \times 10^9$/l. Seven weeks later she was admitted with dental and mouth sepsis and septicaemia. Blood cultures grew a coliform organism and she was treated with broad spectrum antibiotics. Total white cell count on admission was $0.5 \times 10^9$/l with no neutrophils. Sulphasalazine was discontinued and one week later her neutrophil count had risen to $1.3 \times 10^9$/l with clinical recovery.

### CASE 2

A 69 year old man presented with a four week history of severe bloody diarrhoea, weight loss and abdominal pain. Haemoglobin was 12.6 g/dl, white cell count $10.9 \times 10^9$/l with 57% neutrophils, serum albumin 29 g/l and plasma viscosity was elevated at 1.91 cp. A diagnosis of ulcerative colitis was confirmed histologically and a barium enema showed this to be total. He was treated with oral and rectal prednisolone and commenced on sulphasalazine 4 g daily eight days after admission. His course was complicated by a transient ischaemic attack and an arterial embolus to the left foot which improved with conservative measures. He was eventually discharged six weeks after admission taking a reducing course of oral prednisolone, prednisolone enemata, aspirin and sulphasalazine 3 g daily. Six days later he was re-admitted with a gram negative septicaemia, severe oral candidiasis and a perianal abscess, incision of which failed to show pus. Total white cell count was $1.0 \times 10^9$/l with no neutrophils. Despite treatment with antibiotics, granulocyte transfusion and withdrawal of sulphasalazine he died four days later. A postmortem showed quiescent ulcerative colitis.

### CASE 3

A 76 year old woman was admitted with a sudden onset of bloody diarrhoea. Sigmoidoscopy revealed a florid proctitis and radiology and rectal biopsy confirmed the diagnosis of ulcerative colitis. Haemoglobin was 11.0 g/dl and white cell count was $4.5 \times 10^9$/l with 72% neutrophils. Her condition improved with sulphasalazine 4 g daily and prednisolone 10 mg daily on which she was discharged. She also continued her previous drug treatment of imipramine, benzhexol and trifluoroperaxine. She was re-admitted two weeks later with a haemoglobin of 8.7 g.dl, a neutrophil count of $1.1 \times 10^9$/l and 8.2% reticulocytes. Haemolysis continued with reticulocytes rising to 15% and a blood film showing nucleated red cells and spherocytes. A Coombs test was negative and glucose 6 phosphate dehydrogenase levels were within normal limits. Neutrophils fell to $0.25 \times 10^9$/l. Sulphasalazine was discontinued six days after admission and haematological indices returned to normal.

### DISCUSSION

Drug induced neutropenia is uncommon with an incidence in Sweden of 0.009%[4]. This systematic survey showed sulphonamides to be one of the most frequent offenders and responsible for five out of nine fatal cases. There are many reports of sulphasalazine associated agranulocytosis in the literature (5,6,7) but the occurrence of three cases together is highly unusual, although no common link among our patients is apparent. One of the cases also developed haemolysis which has been related to high sulphapyridine levels (8) and slow acetylator status (9). Neutropenia is thought to be due to immunological rather than directly toxic mechanisms (10).

In the treatment of ulcerative colitis new methods are being developed for the delivery of 5-aminosalicylic acid, the active moiety of sulphasalazine, to the colon (11,12) without the presence of the sulphonamide component.

However the active component of sulphasalazine in rheumatoid arthritis is thought to be the sulphonamide moiety (13), so the newer agents developed for ulcerative colitis which exclude this component will be of no value in this condition. If sulphasalazine is to be commonly used in rheumatoid arthritis than a widespread awareness of its potentially lethal side effects is essential. A wise precaution would be to warn all patients to

*(continued on page 83)*

### Adverse Reaction to Sulphasalazine. *(continued from page 73)*

report sore throats or other symptoms of infection immediately during treatment.

### REFERENCES

1. SVARTZ, N. (1942) Salazopyrin, a new sulfanilamide preparation. *Acta. Med. Scand.* **110**, 577–98.
2. PULLAR, T., HUNTER, J. A., CAPELL, H. A. (1983) Sulphasalazine in rheumatoid arthritis: a double-blind comparison of sulphasalazine with placebo and sodium aurothionalate. Br. *Med. J.* **ii**, 1102–4.
3. NEUMANN, V. C., GRINDULUS, K. A., HUBBALL, S., McCONKEY, B. WRIGHT, V. (1983) Comparison between penicillamine and sulphasalazine in rheumatoid arthritis. Leeds-Birmingham trial. *Br. Med. J.* **ii**, 1099–102.
4. ARNEBORN P., PALMBLAD, J. (1982) Drug-induced neutropenia—a survey for Stockholm 1973–1978. *Acta. Med. Scand.* **212**, 289–92.
5. THIRKETTLE, J. L., GOUGH, K. R., READ, A. E. (1963) Agranulocytosis associated with sulphasalazine ('Salazapyrin'). *Lancet* **1**, 1395–7.
6. MADDOCKS, J. L., SLATER, D. N. (1980) Toxic epidermal necrolysis, agranulocytosis and erythroid hypoplasia associated with sulphasalazine. *J. Roy. Soc. Med.* **73**, 587–8.
7. HUTCHINSON, D., WYLD, P. J. (1983) Agranulocytosis associated with sulphasalazine. *New Zealand Med. J.* **96**, 520–3.
8. GOODACRE, R. L., ALI, M. A. M., VANDERLINDEN, B., HAMILTON, J. D., CASTELLI, M., SEATON, T. (1978) Hemolytic anaemia in patients receiving sulfasalazine. *Digestion* **17**, 503–8.
9. VAN HEES, P. A., VAN ELFEREN, L. W., VAN ROSSUM, J. M., VAN TONGEREN, J. H. (1978) Hemolysis during salicylazo sulfapyridine therapy. Am. J. Gastroenterol. **70**, 501–5.
10. HOIGNE, R., LUBBERS, P., GAUTSCHI, M. (1980) Sulphonamide and miscellaneous antibacterial and antiviral drugs. In: Dukes M. N. G., (ed). Meyler's side effects of drugs. Amsterdam: Excerpta Medica 492–523.
11. DEW, M. J., HUGHES, P., HARRIES, A. D., WILLIAMS, G., EVANS, B. K., RHODES, J. (1982) Maintenance of remission in ulcerative colitis with oral preparation of 5-aminosalicylic acid. *Brit. Med. J.* **285**, 1012.
12. WILLOUGHBY, C. P., ARONSON, J. K., AGBACK, H., BODIN, N. O., ANDERSON, E., TRUELOVE, S. C. (1981) Disposition in normal volunteers of sodium azodisalicylate, a potential therapeutic agent in inflammatory bowel disease. *Gut* **22**, A431.

### ACKNOWLEDGEMENT

Miss Victoria Blake for secretarial assistance.

